# Growth performance of broiler chickens fed on sprouted-papaya seed based diets

**DOI:** 10.1080/23144599.2021.1992960

**Published:** 2021-10-28

**Authors:** Sugiharto Sugiharto, Anugrah R. Pratama, Turrini Yudiarti

**Affiliations:** Department of Animal Science, Faculty of Animal and Agricultural Sciences, Universitas Diponegoro, Semarang, Indonesia

**Keywords:** Broiler chickens, growth, carcase, sprouted-papaya seed

## Abstract

The study investigated how sprouted-papaya seed meal (SPSM) a total of affected the growth and carcase traits of broilers. Based on a completely randomized design, 390 day-old Lohmann broiler chicks were allotted to CONT (chicks provided with control diet), SEED25 (diet containing 2.5% papaya seed meal), GERM1 (diet containing 1% SPSM), GERM25 (diet containing 2.5% SPSM) and GERM5 (diet containing 5% SPSM). Body weight and feed intake of chickens were weekly recorded from 14 to 36 days of age, while birds (six birds per treatment group; 30 birds in total) were slaughtered at day 36. Feeding SPSM up to 5% did not impair (*p* > 0.05) broilers’ growth or feed intake. At 5%, SPSM compromised (*p* < 0.05) feed conversion ratio (FCR) and reduced breast meat proportion. SPSM at 2.5% in diets had no (*p* > 0.05) detrimental effects, while papaya seed meal at the same proportion lowered (*p* < 0.05) final body weight, weight gain, and cumulative feed intake. Overall, SPSM may be incorporated in broiler rations up to 2.5% with no harmful effects on growth, feed intake, FCR and carcase traits of broiler chickens.

## 1. Introduction

Yellow maize and soybean meal (SBM) are the primary components of broiler rations, serving respectively as energy and protein sources. During the COVID-19 pandemic, broiler breeders are extremely concerned about the dearth of yellow maize and SBM and their expensive price. Due to lock-down in some regions, the supply chain for yellow maize is interrupted, while the importation of SBM is disrupted during the pandemic. This circumstance forces farmers to seek for alternate energy and protein sources in order to ensure the broiler production sustainability. Papaya (*Carica papaya* L.) seeds represent a waste that is no longer usable. Papaya is one of the most grown tropical crops in the world, with Brazil, India, and Mexico producing the most. On 2016, the global production of papaya fruit was 13.2 metric tons [1]. Given that the seed from ripe papaya accounts for around 16% of the fresh fruit weight [2], papaya seed was produced in 2016 for approximately 2.11 metric tons (in fresh/wet condition). Papaya seed contains a metabolizable energy of 3570 kcal/kg [3] and a protein content of 24–30% [2], making it an excellent alternative broiler feedstuff. Papaya seed has previously been used in broiler diets to decrease the usage of maize and SBM. Due to its high fibre (26.2–45.6%, depending on the papaya varieties) and anti-nutritional compounds (oxalate, tannins, and phytate), papaya seed should however only be included at a maximum of 5%, since greater inclusion levels impaired growth [2,4].

Study documented that germination (sprouting) improved the nutritious content of grains/seeds [5]. Germination enhanced crude protein and crude ash content while decreasing crude fibre in *Moringa* seeds [6]. Moreover, germination modified fibrous fractions in grains, making them easier to digest [7]. Sprouting also decreased anti-nutritional compounds like tannins and phytic acid, allowing the legume seeds to be digested and utilized more thoroughly by animals [8]. So far, there has never been a research on the use of sprouted-papaya seed in broiler diets. The aim of this study was to evaluate how sprouted-papaya seed meal (SPSM) affected the growth and carcase traits of broilers.

## 2. Materials and methods

### 2.1. Production of sprouted-papaya seed meal

The production of SPSM was initiated by collecting the papaya seed (*ca* 50 kg, in fresh/wet condition) from street vendors around the campus. The papaya seed was washed and spread on tray at room temperature for 24 hours. The seeds were soaked for 24 hours, and then placed in perforated bucket. To keep the seed wet during germination, the bucket was sprinkled with water on a daily basis. Papaya seeds were let to germinate for two weeks and then the seeds were collected, sun-dried, and ground (finely ground using a 3 mm screen). Samples of papaya seed and SPSM were obtained for proximate analysis [9]. The results of analysis showed that papaya seed meal had 7.19% moisture, 21.7% crude protein, 22.9% crude fat, 38.7% crude fibre and 10.8% ash on a dry matter basis, whereas SPSM contained 10.3% moisture, 24.7% crude protein, 21.2% crude fat, 36.9% crude fibre and 6.67% ash.

### 2.2. Ethical approval

The Animal Ethics Committee of the Faculty of Animal and Agricultural Sciences, Universitas Diponegoro agreed to the *in vivo* trial (No. 57–06/A3/KEP/FPP).

### 2.3. In vivo trial

A total of 390 day-old Lohmann unsexed broiler chicks were raised with commercial starter feed containing 23% crude protein, 5% crude fibre, 5% crude fat, and 7% ash (according to feed label) from arrival to day 14. From days 14 until 36, the chicks were randomly allotted to CONT (chicks provided with control diet), SEED25 (diet containing 2.5% papaya seed meal), GERM1 (diet containing 1% SPSM), GERM25 (diet containing 2.5% SPSM) and GERM5 (diet containing 5% SPSM). The experiment was arranged according to a completely randomized design (CRD), with five treatment groups and six replicates/pens (13 chicks in each pen; each treatment group consisted of 78 chicks). The chicks were reared in an open-sided broiler house with rice husk bedding. A constant lighting schedule was employed throughout the study. The feeds (in mash form) were formulated to be isocaloric and isonitrogenous and met the Indonesian National Standard for broiler finisher feed [10] (Table 1). On days 4 and 18, the chicks were administered Newcastle disease vaccination by eye drops and drinking water, respectively. On day 12, Gumboro vaccine was also administered by drinking water. Chicks’ body weight (individually weighed), feed intake, and feed conversion ratio (FCR) were recorded weekly. Feed intake was measured as the difference between the amount of feed offered and what was left over, while FCR was computed by dividing of feed intake by the weight gain of broilers. Feed cost per kg live body weight gain and income over feed cost were also determined. At day 36 (after 22 days feeding trial), one male chick per pen (six chicks per treatment group) was slaughtered, defeathered and eviscerated for the assessment of carcase traits of broilers.

**Table 1.** Ingredients and nutritional components of treatment diets (days 14–36)

Items (%, unless otherwise noted)	CONT	SEED25	GERM1	GERM25	GERM5
Yellow corn	58.5	57.0	58.1	57.3	56.1
Palm oil	3.00	3.00	2.90	2.90	2.80
Soybean meal	34.7	33.7	34.2	33.5	32.3
Papaya seed meal	-	2.50	-	-	-
SPSM	-	-	1.00	2.50	5.00
DL-methionine, 990 g	0.19	0.19	0.19	0.19	0.19
Bentonite	0.75	0.75	0.75	0.75	0.75
Limestone	0.75	0.75	0.75	0.75	0.75
Monocalcium phosphate	1.30	1.30	1.30	1.30	1.30
Premix	0.34	0.34	0.34	0.34	0.34
Chlorine chloride	0.07	0.07	0.07	0.07	0.07
Salt	0.40	0.40	0.40	0.40	0.40
Calculated chemical components:					
ME, (kcal/kg)^1^	3000	3000	3000	3000	3000
Crude protein	20.0	20.0	20.0	20.0	20.0
Crude fibre	5.51	6.33	5.83	6.30	7.08
Feed price (IDR/kg)	7,921	7,741	7,836	7,720	7,520

**Key**:^1^ME (metabolizable energy) was calculated based on formula [11]: 40.81 {0.87 [crude protein + 2.25 crude fat + nitrogen‐free extract] + 2.5}

CONT: chicks received control diet, SEED25: diet containing 2.5% papaya seed meal, GERM1: diet containing 1% SPSM, GERM25: diet containing 2.5% SPSM, GERM5: diet containing 5% SPSM

The data were statistically treated according to CRD using analysis of variance (ANOVA, SPSS 16.0 version). The Duncan multi-range test was employed when dietary treatments showed a significant effect (*p* < 0.05).

## 3. Results and discussion

Data in the current study showed that dietary incorporation of SPSM up to 5% did not impair (*p* > 0.05) final body weight, weight gain or cumulative feed intake of broilers (Table 2). From an economical point of view, feeding SPSM up to 5% in the diet, however, exhibited no (*p* > 0.05) economic implications as measured by feed cost per kg live body weight gain and income over feed cost. Different from SPSM, feeding 2.5% of papaya seed meal compromised (*p* < 0.05) body weight (9.15%), weight gain (11.6%) and feed intake (7.69%) when compared to control. This may suggest that sprouting could improve the digestibility and, hence, nutrient utilization of seeds [7,8]. However, this inference should be regarded carefully as feeding SPSM at 5% compromised FCR of broilers. Also, at 5%, SPSM reduced the proportion of broiler breast meat as compared with SPSM fed at 1 or 2.5% (Table 3). Higher fibre content as well as anti-nutritional factors in 5% SPSM-based diet could have reduced feed digestibility resulting in less conversion of feed into body mass. When compared with papaya seed meal, sprouted-papaya seed did not compromise (*p* < 0.05) feed consumption of birds in this study. The improved nutritional qualities (i.e. the increase in crude protein and decrease crude fibre, as mentioned above) and less anti-nutritional factors due to germination [5] seemed to not limit feed consumption of broilers. Yet, we could not provide the actual data on the reduced anti-nutritional compounds due to germination in the current investigation.

**Table 2.** Growth performance of broilers (days 14–36)

Items	CONT	SEED25	GERM1	GERM25	GERM5	SEM	*p* value
Initial BW, g	354	354	353	352	353	3.23	1.00
Final BW, g	1704^a^	1548^b^	1692^a^	1625^ab^	1632^ab^	18.5	0.04
Weight gain, g	1351^a^	1194^b^	1339^a^	1273^ab^	1279^ab^	17.1	0.02
Cumulative FI, g	2249^a^	2076^b^	2311^a^	2241^a^	2406^a^	29.9	<0.01
FCR	1.67^b^	1.74^b^	1.73^b^	1.76^b^	1.88^a^	0.02	0.01
Feed cost per kg live BWG (IDR)^a^	13,203	13,470	13,539	13,602	14,191	143	0.28
Income over feed cost (IDR)^b^	11,157	10,243	10,652	10,334	9,643	225	0.31

**Key**:^a,b^Means with various superscript letters within the same row are significantly different (*p* < 0.05)

^a^Values were taken into account at the time of study as the cost of feed consumed to produce a kg live weight gain

^b^Total revenue minus total feed cost were used to calculate values at the time of the study

CONT: chicks received control diet, SEED25: diet containing 2.5% papaya seed meal, GERM1: diet containing 1% SPSM, GERM25: diet containing 2.5% SPSM, GERM5: diet containing 5% SPSM, BW: body weight, FI: feed intake, FCR: feed conversion ratio, BWG: body weight gain, IDR: Indonesian Rupiah (currency)

**Table 3.** Carcase traits of broilers

Items	CONT	SEED25	GERM1	GERM25	GERM5	SEM	*p* value
Eviscerated carcase (% live BW)	65.5	66.1	68.2	67.8	69.7	0.76	0.44
	% eviscerated carcase						
Breast	36.9^ab^	36.6^ab^	38.9^a^	38.8^a^	33.9^b^	0.56	0.04
Wings	11.9	12.1	10.2	10.9	11.7	0.26	0.12
Thigh	17.7	16.4	15.2	16.3	16.7	0.33	0.18
Drumstick	15.9	16.2	14.6	15.3	14.3	0.25	0.06
Back	17.6	18.7	21.1	18.9	23.4	0.73	0.08
Abdominal fat	1.31	1.33	1.08	1.31	1.30	0.09	0.91
Edible giblets^a^	6.92	6.85	6.34	6.24	6.68	0.19	0.74

**Key**:^a,b^Means with various superscript letters within the same row are significantly different (*p* < 0.05)

^a^Giblets: heart, liver and gizzard

CONT: chicks received control diet, SEED25: diet containing 2.5% papaya seed meal, GERM1: diet containing 1% SPSM, GERM25: diet containing 2.5% SPSM, GERM5: diet containing 5% SPSM, BW: body weight

## 4. Conclusions

Giving SPSM in diets up to a proportion of 2.5% exhibited no negative consequences, but feeding papaya seed meal at the same proportion reduced final body weight, weight gain, and cumulative feed consumption. Feeding SPSM to broiler chickens at 5% of their diet impaired their FCR and breast percentage.
